# Cord Blood Sample Screening for Evidence of Maternal Chagas Disease

**DOI:** 10.3201/eid2304.161287

**Published:** 2017-04

**Authors:** Susan P. Montgomery, Susan L. Stramer

**Affiliations:** Centers for Disease Control and Prevention, Atlanta, Georgia, USA (S.P. Montgomery);; American Red Cross, Gaithersburg, Maryland, USA (S.L. Stramer)

**Keywords:** Chagas disease, cord blood, Trypanosoma cruzi, enzyme immunoassay, radioimmunoprecipitation assay, chemiluminescent immunoassay, parasites

**To the Editor:** The article by Edwards et al. (*1*) contained several errors regarding testing, results, and interpretation of results. The authors incorrectly described the testing performed for the cord blood samples. The American Red Cross (ARC) National Testing Laboratory (NTL) (identified as the “American Red Cross National Donor Testing Laboratory” in the article) has never performed indirect hemagglutination assay testing, a method not licensed by the Food and Drug Administration (FDA) for detection of antibodies to *Trypanosoma cruzi*. In fact, the laboratory used a combination of testing algorithms during 2007–2014, the period of the study, involving 2 different FDA-licensed screening tests and a combination of research and licensed supplemental tests. Each algorithm had varying positive predictive values, ranging from <10% to >50%. The laboratory algorithm from January 2007 to the end of August 2011 included the FDA-licensed Ortho *T. cruzi* enzyme immunoassay (EIA) (Ortho-Clinical Diagnostics, Inc., Raritan, NJ, USA), followed by a research radioimmunoprecipitation assay (RIPA) for supplemental testing of all repeat reactive donations.

On September 1, 2011, the laboratory began using the FDA-licensed PRISM Chagas chemiluminescent immunoassay (Abbott Laboratories, Abbott Park, IL, USA) for donor screening, followed by a combination of RIPA and the Ortho EIA for supplemental testing. On July 30, 2012, the laboratory switched from the RIPA to the FDA-licensed Abbott enzyme strip assay (ESA) (Abbott Laboratories) for supplemental testing, maintaining concurrent testing with the Ortho EIA (*2*). The ARC NTL notified all customers of changes in laboratory testing algorithms.

The results reported in the article do not match those recorded at the ARC NTL. The authors describe 25 samples that tested reactive by indirect hemagglutination and that 19 of those were positive by supplemental RIPA testing. In addition to the incorrect tests described, reported results do not correspond to laboratory records. Reviewing the ARC NTL testing results for the Carolinas Cord Blood Bank facility codes, we found that 34 unique samples tested repeat reactive from October 9, 2007, through October 13, 2014. Of these 34 samples, 11 were positive on supplemental RIPA testing and none were ESA positive; testing with RIPA or ESA was dependent on the algorithm in place at the time. Of the 11 samples that were reactive in screening tests and showed positive results in supplemental tests, 2 positives were identified from October–November 2007, 1 from November 2008, 5 from June 2009–January 2010, 2 from May–June 2010, and 1 from 2012 (which does not correspond to the data in the figure or patterns described in the discussion). An additional 4 screening test repeat reactive donations were tested during 2015–2016, with 1 ESA positive but Ortho EIA nonreactive.

Test results of submissions from other facility codes for Duke University were reviewed to see whether any positive samples were submitted from a different North Carolina laboratory; we found 10 additional screening test repeat reactive donations, but none had positive results by supplemental testing. We do not know whether testing of cord blood samples was performed by another laboratory; only the ARC NTL was described in the methods of this study.

Much of the interpretation of results was misleading. The authors considered any screening test positive result as being sufficient for confirmation of infection. To be meaningful, all samples with reactive results should be tested further, and only those with reactive or positive results by at least 2 different tests considered for any investigation of epidemiologic trends. Furthermore, a single serologic screening test reactive result confirmed as positive, though useful for blood donor management, does not define a confirmed diagnosis of Chagas disease (*3*).

The authors’ use of the term incidence does not agree with the epidemiologic definition of that term. The authors state, “The incidence of confirmed Chagas disease among mothers who donated their neonate’s cord blood varied over time,” “The incidence of Chagas disease varied over time,” and “A strength of this study is its large sample size, particularly because the incidence of this disease is low.” However, no incident *T. cruzi* infections were identified by their study. No evidence of acute infection was presented. All mothers who donated cord blood were chronically infected; the testing of their samples revealed the prevalence of positive results among the samples tested in a given period (had the numbers used been accurate, which they were not). This distinction is key because acute infections are more likely to be transmitted through blood transfusion and patients’ infections are more likely to be successfully cured by antitrypanosomal treatment during the acute phase of infection, before development of cardiac manifestations.

Preventing and controlling congenital Chagas disease is a serious public health issue; the screening of mothers at risk for transmitting *T. cruzi* infection to their babies is considered key to accomplishing these factors. The evidence base to support screening recommendations must be high-quality and accurate. Other studies have emphasized this risk in the US population, particularly in Latin American immigrant mothers (*4*), but further evidence is needed to guide policy recommendations. The report of Edwards et al. (*1*) could be a contribution to this needed evidence base, but only if reported data are accurate and appropriately interpreted.
